# Improving Methane Emission Estimates from Nonsewered Wastewater during Storage in Septic and Holding Tanks

**DOI:** 10.1021/acs.est.5c17325

**Published:** 2026-05-12

**Authors:** Kelsey Shaw, Charles B. Niwagaba, Linda Strande, Caetano C. Dorea

**Affiliations:** † Department of Civil Engineering, 8205 University of Victoria, Victoria, British Columbia V8P5C2, Canada; ‡ Sandec: Department of Sanitation, Water and Solid Waste for Development, 28499 Eawag: Swiss Federal Institute of Aquatic Science and Technology, Dübendorf 8600, Switzerland; § College of Engineering, Design, Art and Technology, Makerere University, P.O. Box 7062, Kampala, Uganda

**Keywords:** emission factors, greenhouse gas inventories, methane correction factors, population equivalents, intergovernmental panel on climate change, fecal sludge management

## Abstract

This study quantified methane (CH_4_) emissions from nonsewered sanitation (NSS) in septic and holding tank containments for onsite storage of wastewater. It further examined how the measured emissions and corresponding parameters relate to the Intergovernmental Panel on Climate Change (IPCC) approach, which uses Tier 1 default parameters and simplified assumptions that do not capture the variability of NSS containment conditions. Paired with questionnaires to residents, samples were collected from household containments in rural Southern Coastal British Columbia (BC), Canada (n = 16), and urban Kampala, Uganda (n = 17). Gas samples and 22 physicochemical parameters were quantified *in situ* or in a laboratory. Median net cumulative CH_4_ concentrations were slightly higher in BC (20,150 ppm) than in Kampala (14,950 ppm), despite less favorable conditions for biological activity. Scum depth exhibited the strongest correlation with CH_4_ concentrations (BC r_s_ = 0.76; Kampala r_s_ = 0.88). Compared to Tier 1 IPCC estimates for Canada (18.0 g CH_4_ capita^–1^ day^–1^) and Uganda (11.1 g CH_4_ capita^–1^ day^–1^), median emissions based on CH_4_ measurements were 30.8 and 12.6 g CH_4_ capita^–1^ day^–1^ for Southern Coastal BC and Kampala, respectively, when normalized by the number of household residents, and 82.3 and 85.8 g CH_4_ m^–2^ day^–1^ when normalized by the surface area of the containment. Methane correction factors (MCFs) calculated in this study were different from those recommended by the IPCC. These results reveal important limitations in the IPCC methodology for scaling point-source measurements using current Tier 1 defaults, including the difficulty of reliably estimating organic loading values and population equivalents, and the inadequacy of IPCC MCF values to represent highly variable and context-specific conditions in containments.

## Graphical abstract

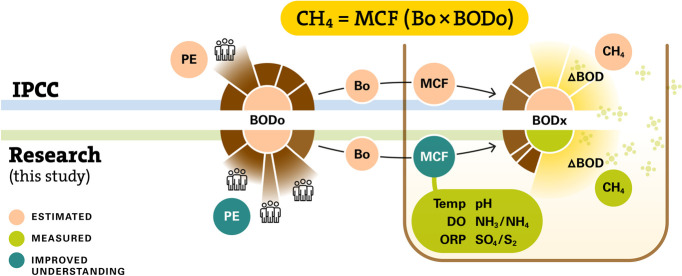

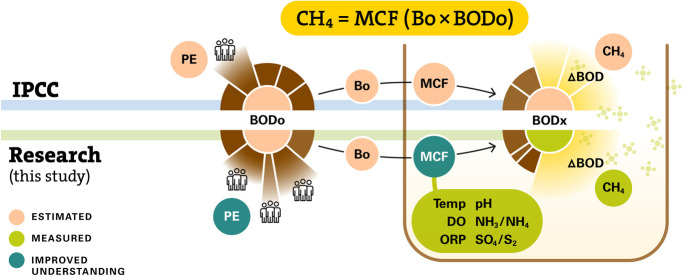

## 1 Introduction

Nonsewered sanitation (NSS) is essential to meeting the sanitation needs of billions of people. Indeed, 58% of the global population is not connected to sewers.^1^ Reliance on NSS spans dense urban neighborhoods in low- and middle-income countries (LMICs), where NSS can account for 60–90% of sanitation systems, to rural communities in high-income countries (HICs), where 5–30% rely on onsite systems.^1,2^ In this context, some of the most widely used forms of storage for onsite wastewater in NSS are septic tanks (i.e., multichamber tanks connected to leach fields) and holding tanks (i.e., commonly single-chamber tanks connected to soak pits).^1,2^ These NSS storage containments often represent the first improved service level relative to open defecation.^3,4^ To achieve safely managed sanitation, such containments form part of a broader service chain that includes collection and transport to treatment.^5^ However, the sustainability of these services is constrained by technical, financial, and regulatory challenges that are inherent to each region and produce great variability,^6^ with heterogeneous containment designs and siting,^7,8^ variable influent composition and volumes (e.g., black water only vs mixed black/gray water)^9,10^ and differences in access for emptying and safe transport.^5,11^ These realities shape both performance and environmental outcomes, including greenhouse gas (GHG) emissions.

Methane (CH_4_) is biologically generated in NSS containments and is a potent GHG, as its 100-year global warming potential is 28 times that of carbon dioxide.^12^ Understanding CH_4_ emissions emitted through NSS containments is therefore essential to plan climate-conscious sanitation and help meet global development commitments. To this end, the Paris Agreement requires countries to report CH_4_ emissions as part of their national inventories.^13^ The need for reliable CH_4_ estimates from NSS containments also aligns directly with United Nations Sustainable Development Goals (SDGs) 6 (Clean Water and Sanitation) and 13 (Climate Action).

Currently, the governing methodology to generate estimates of GHGs from NSS is outlined in the Intergovernmental Panel on Climate Change (IPCC) inventory guidelines.^14^ The IPCC method posits that CH_4_ emissions are based on population equivalents (PEs), which determine organic loading rates for each region, presented as per capita biochemical oxygen demand (BOD) and influent biochemical oxygen demand (BOD_0_); the maximum CH_4_-producing capacity for domestic wastewater (B_0_) based on the stoichiometric conversion of glucose; and system-specific methane correction factors (MCFs) that reflect assumptions about anaerobic conditions. By definition, the MCF should represent the *in situ* conditions that facilitate or inhibit anaerobic degradation, such as dissolved oxygen (DO) gradients, redox conditions, inhibition by free ammonia, or nutrient limitations.^15–17^ However, the current IPCC guidelines rely on proxy classifications for MCFs, such as “septic tank” or “pit latrine”. For septic tanks, which the guidelines do not define as being connected to a leach field or not, they only cite three U.S.-based sources.^18–20^ Notably, the default MCF value of 0.5 prescribed in the guidelines substantially exceeds the mean value of 0.22 reported in these three studies,^18–20^ highlighting both a significant lack of regional representativeness in the available field evidence and a disconnect between empirical data and the values recommended in these guidelines. The IPCC methodology estimates CH_4_ emissions as follows:

$${\mathrm{CH}}_{4}({\mathrm{g CH}}_{4}{\mathrm{capita}}^{\mathrm{-1}}{\mathrm{day}}^{\mathrm{-1}})=\mathrm{MCF}\;(\mathrm{unitless})\times {\mathrm{BOD}}_{0}({\mathrm{g BOD capita}}^{\mathrm{-1}}{\mathrm{day}}^{\mathrm{-1}})\times B_{0}({\mathrm{kg CH}}_{4}/\mathrm{kg BOD})$$

The IPCC methodology, therefore, scales up GHG emissions using estimated influent organic loading and a steady-state model approach.^14^ However, it uses Tier 1 default parameters and relies on assumptions that do not fully reflect microbial degradation dynamics in NSS,^21^ and on PE-based loadings that can misrepresent actual toilet use and wastewater generation.^22^ All field-based studies that have directly measured CH_4_ emissions from NSS containments with stored wastewater (i.e., septic tanks and holding tanks) have concluded that the current guidelines should be updated with field-derived data.^18,20,23–26^ Unfortunately, although the number of publications on NSS-related GHG emissions has increased dramatically in the last 20 years,^27^ these studies have remained scarce and have covered very few countries (i.e., Ireland, USA, and Vietnam).^18,20,23–26^

To address these issues, we applied a field-based method to quantify CH_4_ emissions and a comprehensive suite of physicochemical parameters from NSS containments in two contrasting settings: rural Southern Coastal British Columbia (BC), Canada, and urban Kampala, Uganda. These sites are used to explore the limitations in the current methodological framework rather than to represent annual or global emission patterns. This study had four specific objectives: (1) directly measure CH_4_ emissions and generate field-based CH_4_ emission estimates from NSS containments; (2) contrast these values in two very different regions and usage scenarios; (3) identify potential proxies for scaling CH_4_ emissions; and (4) evaluate how accounting for usage patterns influences reported emissions. This provides a comprehensive assessment that reveals limitations in the current Tier 1 IPCC methodology, which is not based on primary data collection, and highlights pathways to improve the accuracy, scalability, and policy relevance of NSS-containment-related CH_4_ emission estimates.

## 2 Materials and Methods

### 2.1 General Overview

We investigated CH_4_ emissions from household containments, with data collected from 16 single-family homes in rural Southern Coastal BC in April–June 2023, and 17 multifamily homes in urban Kampala in January–February 2024, following a convenience sampling protocol. Data were categorized into three main types: (i) questionnaire (i.e., demographics, technical specifications, and maintenance practices); (ii) *in situ* field measurements of physicochemical parameters and gas samples; and (iii) laboratory analysis of additional physicochemical parameters of stored wastewater.

In this study, “stored wastewater in containment” refers to all fractions of wastewater inside a septic or holding tank, which could include more liquid fractions, a floating scum layer, and thicker fractions comprising more settled solids. The term “wastewater depth” refers to the combined depth of all layers. Influent wastewater sources include toilet wastewater (from cistern- or pour-flush toilets) and can also include bathing water (from showers and personal hygiene activities), laundry water (from hand or machine washing), and kitchen greywater (from sinks or similar sources). Containments in BC broadly represent typical NSS in Canada.^28,29^ While the core urban areas rely on municipal sewers, approximately 20% of all BC homes use septic tanks.^30^ We sampled from a subset of NSS containments (i.e., holding tanks) in Kampala, Uganda. Kampala, Uganda’s capital and largest city, has a population of nearly 1.8 million,^31^ where approximately 90% of the residents rely on NSS,^32^ with an estimated 15–30% using holding tanks.^33,34^ Seasonal variability was not considered, although sampling periods captured typical conditions in both study sites (details in Supplementary Methods S3). Importantly, using consistent sampling procedures from similar containments over short-term campaigns allowed us to compare containment characteristics across the two geographical locations.

### 2.2 Questionnaire about Usage and Physicochemical Parameters of Containments

Questionnaires were used to collect information on containment design, usage patterns, and emptying frequency. Ethical approval was obtained from the University of Victoria (Protocol No. 22-0742), and the Uganda National Council for Science and Technology (Reg. No. SIR275ES), and the Swiss Federal Institute for Aquatic Science and Technology (Eawag) (Policy Directive 16-09). Survey questions and eligibility criteria are available in the Supporting Information (SI) (Supporting Methods S4).

Physicochemical parameters were measured *in situ* or in the laboratory. *In situ* measurements included DO, temperature, pH, oxidation–reduction potential (ORP), electrical conductivity (EC), containment and wastewater depth, surface area, and volume. Sampling was conducted at three depths in the containment (top, middle, and bottom) to capture vertical variability. Variables analyzed in the laboratory were chosen based on established links to anaerobic degradation and CH_4_ production or inhibition.^21,35,36^ They included chemical oxygen demand (COD), soluble COD (sCOD), BOD (5-day), total organic carbon (TOC), total solids (TS), total suspended solids (TSS), volatile solids (VS), volatile suspended solids (VSS), silica content, total nitrogen (TN), ammonium, nitrite, nitrate, total phosphorus (TP), orthophosphate, and sulfide. Standardized quality assurance and control (QA/QC) protocols were followed based on established wastewater and fecal sludge methodologies.^35,36^ A full list of measured parameters, instruments and tools used, transport procedures, triplicate sampling rates, and error margins is available in the SI (Supporting Information Methods S1 and Tables S19 and S20).

### 2.3 Methane Emission Measurements and Estimation Calculations

Gas samples were collected using a modified floating flux chamber, adapted for consistency across field conditions, to capture CH_4_ concentrations, including accounting for multiple chamber and single chamber configurations (Supporting Information Methods S2 and S3). It should be noted that the boundary condition of this comparison was the tank itself and did not extend to onsite or offsite treatment units (i.e., leach field, soak pit, or road-based transport to treatment). A minimum of five grab samples were collected over 30 min, with emission rates validated by linear CH_4_ release trends (Table S21 and Figures S87–S125). Samples were stored and shipped in vacuum-sealed 12 mL glass vials and analyzed using gas chromatography with flame ionization (GC-FID) and electron capture (GC-ECD) detectors (2010 Shimadzu, Germany) at Eawag laboratories (Kastanienbaum, Switzerland). QA/QC methods, analytical protocols, and linear trend data are available in the Supporting Information S3. CO_2_ concentrations were measured and correlated linearly with CH_4_ trends (Figure S126). N_2_O was not detectable across all sampled containments, consistent with results from previous NSS field studies,^18,24^ and therefore is not discussed further.

Emission rates were expressed in three ways: as cumulative concentrations measured during 30-min sampling periods (in parts per million—ppm), as per capita rates based on PEs (g CH_4_ capita^–1^ day^–1^), and as rates normalized to the surface area of each containment (g CH_4_ m^–2^ day^–1^). All analytical procedures and equations used in deriving emission rates per capita, and per containment, are described in full in the Supplemental Methods S3. Importantly, we used two methods to estimate PE. The first estimate of PE was consistent with current IPCC methodology^14^ and existing NSS literature,^18,20,24,25^ whereby one recorded user equated to one PE. For the second approach, PEs were adjusted based on estimates of the time that individuals spent at each location, thereby potentially better reflecting actual wastewater contributions (Table 1). The relationship between time spent at home and organic loading is complex and context-specific which is one of the reasons PEs are notoriously hard to determine for individual toilets in nonsewered areas. In high-income settings, cooking, eating, bathing, and using the toilet occur more often at home.^37^ However, this pattern shifts in low-income urban contexts, where high mobility^38,39^ and limited access to improved facilities^40^ can result in eating more food and using more toilets outside the home. These adjusted values served as proxies solely to demonstrate sensitivity to time-activity assumptions and their resulting comparative outcomes (refer to Table 1 footnotes). Further detailed analysis beyond this study’s scope would be needed to confirm these values.

**Table 1:** Overview of Population Equivalent Calculation Methodologies

Area	Establishment Category	Category Type	IPCC Method	Adjusted for Time Spent at Home^a^
Southern Coastal BC	Household	Single Family	1 PE = 1 Resident	1 PE = **0.7** x Residents
Kampala	Household	Multi-Family		1 PE = **0.3** x Residents

a People are estimated to spend 70% of their time at home in the Netherlands, a similar socioeconomic context to Southern Coastal BC,^37^ and for comparative purposes, 30% was chosen for Kampala based on the literature.^38–40^

### 2.4 Statistical Analysis

Statistical analyses were conducted in R (v4.4.1).^41^ Data visualization was performed using the tidyverse suite, including ggplot2 (v3.5.1) and dplyr (v1.1.4).^42^ Normality was assessed for each variable using the Shapiro-Wilk test.^43^ All data were non-normally distributed, with the exception of pH, which is a log-transformed metric; therefore, for consistency, nonparametric (Wilcoxon rank-sum) methods were used to evaluate significant differences between variables.^44,45^ A significance threshold of *p* < 0.05 was applied throughout, and Fisher’s exact test was used for categorical variables due to small sample sizes (Table S6a).^46^ We contrasted results derived from Southern Coastal BC vs Kampala for all parameters (Table S6a and S11). Correlation analyses between each questionnaire and physicochemical parameter and CH_4_ measurement were conducted separately for each location to account for statistically significant differences between the two locations, as confirmed by multivariate analyses (Statistical Analyses S6b–d; see Results and Discussion Section 3.1.2). Spearman’s rank correlation was used to identify statistically significant associations between parameters, with strong correlations defined as |r_s_| > 0.7 and moderate correlations as 0.5 < |r_s_| < 0.7 (Table S18).^47^ As physicochemical parameters measured at the top, middle, and bottom of the containment showed no statistically significant differences (Table S11; see also Results and Discussion section 3.1.2), average values across depths for each containment, along with questionnaire data, were used for correlation analysis with measured CH_4_ emissions. CH_4_ emissions data were highly variable and non-normally distributed, and as such, were assessed using several central tendency metrics, with the median selected as the most representative value for reporting (Figures S129 and S130). For correlation analyses, cumulative CH_4_ emissions over the 30-min sampling period were used rather than normalized values, to preserve relationships with scale-dependent variables. While flux rates are standard practice in sewered sanitation, their application in NSS is still being transferred, with underlying assumptions remaining insufficiently validated. Importantly, an objective of this study was to illustrate how methodological norms may obscure real variation in observed emissions. All statistical workflows and results are publicly available in the Eawag Research Data Institutional Collection (ERIC) repository (10.25678/000FPN), adhering to the FAIR data principles of transparency and reproducibility.^48^

## 3 Results and Discussion

### 3.1 Questionnaire, *In Situ,* and Laboratory Analysis

#### 3.1.1 Physical Characteristics of Containments and Usage Habits

Containment characteristics (i.e., types of influent wastewater, containment dimensions) and context information (i.e., building usage, number of users, and ownership) revealed regional specificities (Table 2), with detailed results by containment and depth, as well as statistical analyses available in Tables S3–S5, S9–S11 and Figures S2–S37. Containments were smaller in Southern Coastal BC than in Kampala (median volumes of 1.9 m^3^ vs 8.6 m^3^, respectively) and served fewer occupants (2 vs 16). The containments in Southern Coastal BC included baffled septic tanks followed by leach fields, whereas in Kampala, containments were primarily single compartment holding tanks followed by soak pits. This confirmed that variability in containment configurations and usage patterns depends on regional location.^18,20,24,25^ For example, baffled septic tanks with leach fields were also observed more frequently in the USA^18,20^ than in Vietnam, where households were mainly equipped with containments receiving black water, without leach fields, going to open drains or soak pits.^24,25^

**Table 2:** Overview of Sampled Containments. Entries Detail Building Usage, Types of Influent Wastewater, Containment Dimensions, Number of Residents and Ownership (Median and Range Values Where Applicable), with Comparisons to Literature^a^,^b^,^c^,^d^

Parameter	Southern Coastal BC	Kampala	Literature
Type of Establishment	Household	Household	Household
Building Usage	Single Family (*n* = 16)	Multi-Family (*n* = 17)	Single Family (*n* = 8,^18,20^ 10,^24^ 23 ^25^)
Ownership	Owned	Rented	–
Configuration & Discharge	Baffled with Leach Field	Unbaffled with Soak Pit	Baffled^18,20,24,25^ Leach field^18,20^
Influent Wastewater	Blackwater and Greywater	More Instances of Black Water Only	Blackwater & Greywater^18,20^ Blackwater only^24,25^
Emptying Practices	Fully Emptied	Partially Emptied	–
Cleansing Material	Toilet Paper	Toilet Paper or Water	–
Toilet Type	Cistern Flush	Cistern and Pour-flush	Cistern flush^18,20,24,25^
Solid Waste**^d^**	None Observed	Frequently Observed	–
Surface Area (m^2^)	**0.9** (0.2–2.8)	**3.3** (0.7–11.6)	–
Containment Depth (m)	**1.8** (1.2–5)	**3.0** (1.6–4.5)	–
Containment Volume (m^3^)	**1.9** (0.3–5.3)	**8.6** (1.6–46.3)	4.5–5.7 ^18^
			2.8–4.7 ^20^
			1.6–3.0 ^24,25^
Wastewater Depth (m)	**0.7** (0.3–4.9)	**0.8** (0.3–1.9)	–
Wastewater Volume (m^3^)	**1.5** (0.2–4.2)	**6.9** (1.3–40.3)	–
Emptying Interval (Years)	**5** (2.5–15)	**2** (0.3–6)	–
Last Emptied (Years before Sampled)	3 (0.3–11)	3 (0.3–10)	1–>18 ^18^
			0.5–>12 ^20^
			7–>19 ^24^
			3.9–23 ^25^
Residents	**2** (2–4)	**16** (4–88)	2–6 ^18,20,24,25^
Scum Depth (cm)	8.0 (0–12)	3.0 (0–8.0)	0–13 ^18^
			2.1–7.5 ^20^

a Statistically significant differences between Kampala and BC are shown in bold (*p* < 0.05).

b Literature values are field-based emission studies of similar NSS containments to this study.

c Only the statistically significant questionnaire parameters are shown in the table (*p* < 0.05, Fisher’s Exact test).

d Nonorganic municipal solid waste; identified by visual inspection.

#### 3.1.2 Stored Wastewater Characteristics

The characteristics of stored wastewater in this study are summarized by region (Table 3) and by containment and depth within containment (Tables S7 and S8 and Figures S38–S83). We found no statistically significant differences across depths (top, middle, bottom) in any measured physicochemical parameters (Table S11), and we therefore aggregated values and present averages in Table 3. This result contrasts with the common assumption that stored wastewater stratifies into distinct layers (e.g., solids, liquid, scum),^2,35^ which was observed in household containments from Vietnam, with increasing concentrations of solids and organic matter with depth.^25^ Our contrasting results may be due to continual fresh inputs, creating hydraulic disturbances and limiting solids settling. Overall, our results confirm the variable nature of the effects of depth on stored wastewater characteristics and question whether three-layer stratification consistently occurs in septic and holding tanks.

**Table 3:** Physicochemical Characteristics of Stored Wastewater in Containments in Southern Coastal BC and Kampala^a^

			*In Situ* analysis				
Area	Establishment Category	Category Type	Temp (°C)	pH	DO (mg/L)	ORP (mV)	EC (μs/cm)
Southern Coastal BC	Household	Single Family (*n* = 16)	**15** (9–24)	6.9 (6.4–7.3)	**0.8** (0.2–7.8)	**–216** (−15 to −414)	**1,203** (303–2,158)
Kampala	Household	Multi-Family (*n* = 17)	**24** (23–26)	6.9 (6.5–7.4)	**0.1** (0.1–0.5)	**–280** (65 to −470)	**1,532** (644–6,767)
GHG-focused field studies^18,24–26^	Household	Single Family	9–31	3.7–8.5	0.02–9.8	–150 to −563	–
General onsite stored wastewater characterization studies^9,10,21,49–51^	Varies	Varies	10–40	5.7–8.9	–	–	1,300–15,400

Laboratory analysis								
TS (g/L)	VS (%)	TSS (g/L)	VSS/TSS	Silica Content (g/L)	COD (mg/L)	sCOD (mg/L)	BOD (mg/L)	TOC (mg/L)
2.6 (0.4–14.7)	**72** (33–95)	1.8 (0.1–10.4)	**0.91** (0.73–0.98)	0.09 (0.00–0.50)	4,817 (573–21,920)	563 (263–1,763)	**490** (23–1,755)	**359** (45–1,209)
29.9 (0.5–98.8)	**46** (26–72)	6.5 (0–28.9)	**0.78** (0.53–1)	13.27 (0.00–43.93)	18,429 (360–56,292)	739 (168–7,840)	**1679** (137–5,440)	**1,449** (60–2,680)
–	–	5.2–10.9	–	–	5,190–29,592	–	0–33,422	–
0.002–121	4.3–84.9	0.1–107.8	0.08–1	0–72% of TS*	375–91,850	5,760–13,500	57–11,207	–

Laboratory analysis							
TKN (mg/L)	TN (mg/L)	Nitrate (mg/L)	Nitrite (mg/L)	Ammonia (mg/L)	Total Phos (mg/L)	Ortho-Phos (mg/L)	Sulfide (mg/L)
**152** (14–581)	**157** (19–586)	5.0 (5.0–16.2)	**0.03** (0.01–0.12)	**86** (2–140)	30.3 (6.1–63.7)	**30.5** (6–61.6)	**1.94** (0.15– 13.40)
**772** (25–2,508)	**773** (152–2,526)	10.8 (0.15–182.8)	**0.13** (0.01–0.38)	**220** (83–1,158)	130.5 (8.8–505.6)	**14.6** (4.8–180.4)	**0.03** (0.00– 9.91)
–	1.9–2,200	0–56.7	–	0.7–750	69–521	–	–
–	–	–	–	0.4–1,963	–	–	–

a A total of 22 parameters were analyzed either *in situ* or in the laboratory. Data shown are composite median and range values where applicable, with comparisons to literature. Statistically significant differences between Kampala and Southern Coastal BC are shown in bold (*p* < 0.05, Wilcoxon test).

We found statistically significant differences between Southern Coastal BC and Kampala for 14 of the 22 parameters examined (Table 3). In Kampala, stored wastewater had higher *in situ* temperature, EC, BOD, TOC, TKN, TN, nitrite, and ammonia, lower concentrations of DO, and more reducing conditions (i.e., ORP), likely due to a higher fraction of the tanks receiving only blackwater instead of mixed blackwater and graywater (Table 2). In contrast, Southern Coastal BC containments had higher fractions of VS and VSS/TSS as well as sulfide concentrations. All values fell within, or at the lower end of the highly variable ranges reported in prior studies,^9,10,18,21,24–26,35,49–51^ aligning with previous research that consistently reports high variability depending on region and non-normal distributions in physicochemical parameters.

#### 3.1.3 Cumulative Methane Emissions and Potential Proxies

Median cumulative CH_4_ emissions over 30 min from Southern Coastal BC containments (20,150 ppm) were 35% higher than those in Kampala (14,950 ppm), but the difference was not statistically significant (Figure 1). Studies investigating comparable containment types in Vietnam reported similar ranges (median emissions of 11,420 ppm after 40 min^25^ and 14,000 ppm after 60 min^24^).

**Figure 1:**
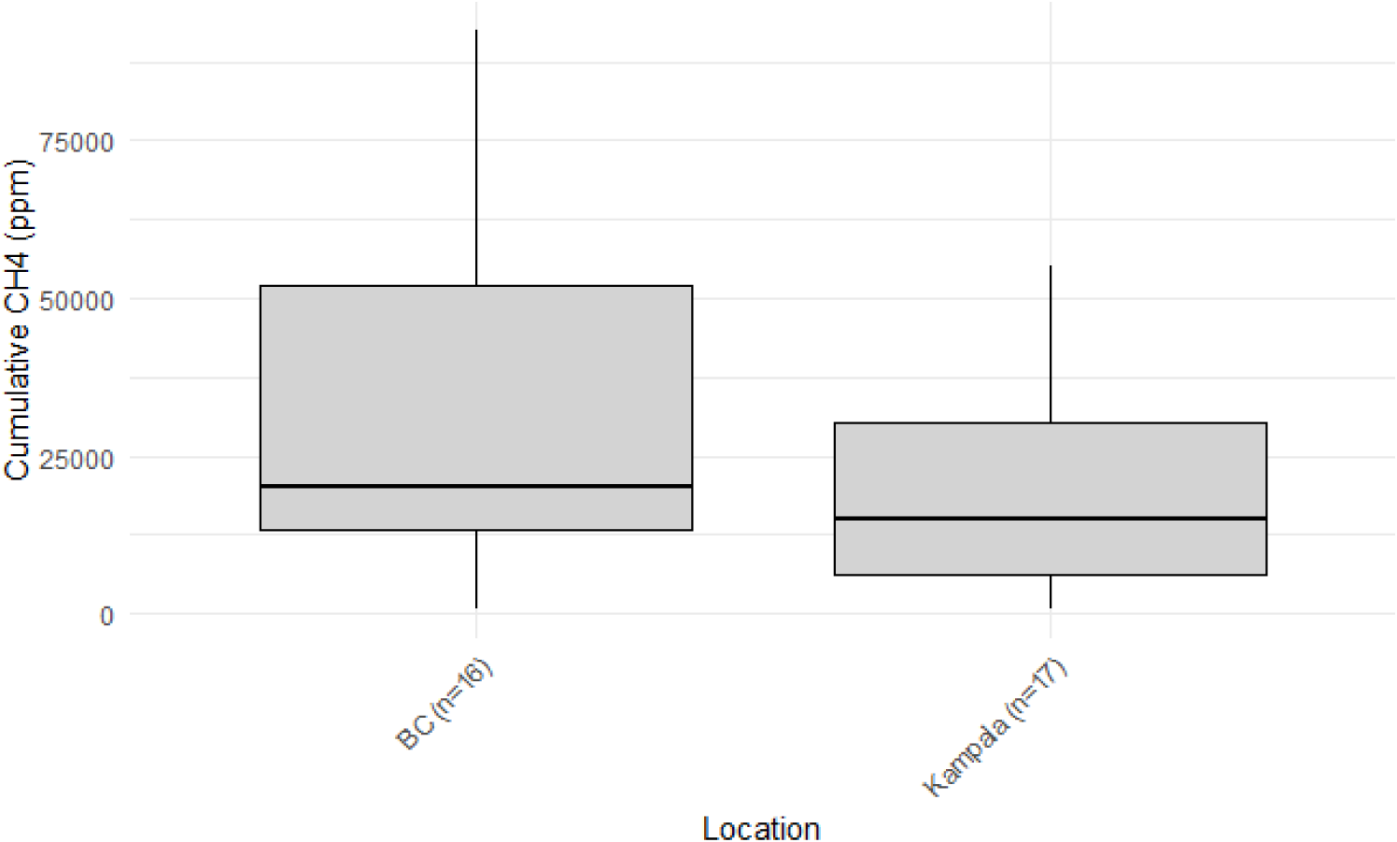
Net accumulated household CH_4_ emissions (in ppm) in the flux chamber after 30 min in Southern Coastal BC and Kampala. Boxes represent interquartile ranges (IQRs), and lines indicate median values.

We evaluated correlations between net cumulative CH_4_ emissions on one hand and total and biodegradable organics (BOD, COD, sCOD, VS/TS, and VSS/TSS) on the other and found no statistically significant relationships at either study site, along with all other measured physicochemical parameters (Table S18). When examining other variables, scum depth had the strongest statistically positive correlation with net cumulative CH_4_ emissions in both Southern Coastal BC (r_s_ = 0.76) and Kampala (r_s_ = 0.88) (Table 4). One explanation could be that the scum layer, typically composed of fats, oils, and greases (FOGs),^52^ provides a biodegradable substrate for methanogens.^53^ Research on anaerobic lagoons has also shown that biogas production can lead to buoyant sludge and scum formation.^54^ While causation is not demonstrated, these results suggest that further investigation into the mechanistic drivers could reveal that (i) biological activity generates gas bubbles, which help lift FOGs to the surface and contribute to scum accumulation, and (ii) scum buildup may be both a contributor to and an indicator of anaerobic activity.

**Table 4:** Correlation between Measured Net Cumulative Methane after 30 Min and Metadata Parameters in Southern Coastal BC and Kampala^a^,^b^,^c^

Parameter	Kampala *r*_s_	*p*-value	Southern Coastal BC *r*_s_	*p*-value
Number of Residents	0.54	<0.05	–	–
Total Wastewater Depth	–0.63	<0.01	–	–
Scum Depth	**0.88**	**<0.001**	**0.76**	**<0.001**
Time since last emptied	–0.65	<0.01	–	–

a Strong correlations |r_s_| ≥ 0.7 and *p* < 0.001 are shown in bold.

b Values are Spearman rank correlation coefficients (r_s_) between measured net cumulative CH_4_ after 30 min and questionnaire, *in situ*, and laboratory-measured parameters.

c Only medium or strong correlations (|r_s_| ≥ 0.5) that were statistically significant (*p* < 0.05) are shown. Full results are available in Table S18.

In Kampala, CH_4_ emissions were positively correlated with the number of residents (r_s_ = 0.54) and negatively correlated with wastewater depth (r_s_ = −0.63) and time since last emptied (r_s_ = −0.65), defined as the interval between the last emptying and our sampling (Table 4). It is likely that the higher number of residents and primarily blackwater-only influent (Table 2) resulted in frequent inputs of more concentrated organic and nutrient wastewater in Kampala (Table 3). This, in turn, may have led to more frequent emptying, producing less full containments at the time of sampling, which are patterns that could be confounded with regional differences between Southern Coastal BC and Kampala. Univariate analyses identified both the number of residents and influent wastewater type as significant predictors for Kampala containments, while multivariate analyses confirmed these regional effects (region explained up to 76% of multivariate variation; Tables S6b-d). However, since these are single-point measurements, additional data are needed before generalizing the role of tank volume or emptying frequency in predicting emissions. In addition, one should not assume that the number of household residents is equivalent to PEs (and, by extension, BOD_0_), as people use multiple toilets in many locations throughout the day.^39^ We did not take direct measurements of organic influent loadings, which are needed to confirm this. Further, due to logistical constraints, previous studies have often only sampled at the time of emptying. For example, two studies in Vietnam exclusively sampled from containments scheduled for emptying,^24,25^ while in this study, we also included recently emptied containments. This difference may explain why Moonkawin et al., observed greater CH_4_ emissions in containments correlated with greater sludge depth (i.e., settled solids),^25^ while in our study, shallower total wastewater depths were associated with higher CH_4_ emissions. Overall, our results indicate that in Kampala, CH_4_ emission patterns are sensitive to both recent usage dynamics and influent wastewater characteristics. Moreover, the containment type and the time frame when sampling occurs can strongly shape the observed relationships; hence, extrapolations about drivers of CH_4_ emissions must be made with caution.

### 3.2 The IPCC Steady-State Model: Limitations and Biases

#### 3.2.1 Influence of Organic Loading Rates

Figure 2 illustrates the assumptions made in the IPCC steady-state model, in comparison to the analytical results of wastewater stored in containments in this study. In both approaches, BOD_0_ loadings are assumed on a per capita (PE) basis, which is multiplied by its maximum CH_4_-producing capacity (B_0_ = 0.6 kg CH_4_ per kg BOD) and an MCF to estimate resultant CH_4_ emissions. The IPCC methodology is a simplified approach that relies on estimated or default parameters in the absence of field data, while our research approach incorporates direct measurement and understanding of actual *in situ* environmental conditions.

**Figure 2:**
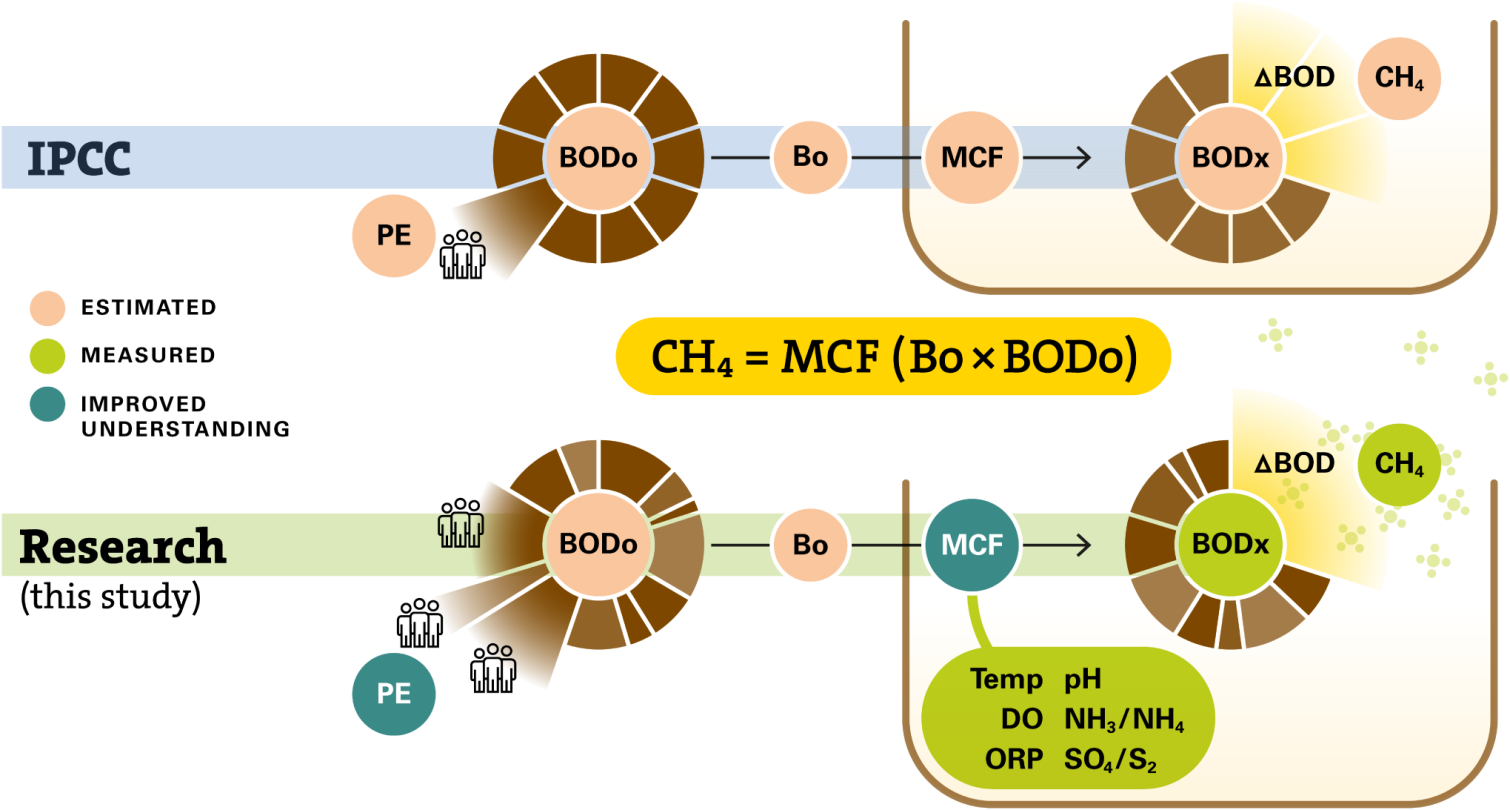
Conceptual illustration comparing the steady-state model and governing equation used by the IPCC to estimate methane (CH_4_) emissions with the enhanced field-based approach developed in this study. Acronyms: BOD_0_ – influent biochemical oxygen demand; B_0_ – ultimate methane-producing potential; MCF – methane correction factor; CH_4_ – methane; PE – population equivalent; ΔBOD – change in BOD; BOD*_x_* – *in situ* BOD concentration in containment; DO – dissolved oxygen; NH_3_/NH_4_ – ammonia/nitrogen; ORP – oxidation–reduction potential; SO_4_^2 –^ – sulfate; S_2_^–^ – sulfide.

The IPCC conceptual model provides a useful baseline for discussion. However, each of the components carries some degree of inaccuracy, including organic loading rates. Indeed, the default value of B_0_ is the stoichiometric, complete decomposition of glucose (C_6_H_12_O_6_), and the BOD_0_ values are derived from a limited data set. To convert COD to BOD, the IPCC model applies an empirical COD:BOD ratio of 2.4 for domestic wastewater;^14^ however, the associated BOD_0_ values are based on a data set from industrial wastewater plants in one region of the USA.^14,55,56^ This is problematic because the characteristics of nonsewered wastewater are highly variable,^35^ and influent organic loading is difficult to measure or predict for individual containments due to diverse usage patterns.^57,58^ *In situ* median COD:BOD_5_ ratios in our study (4.1–9.4) were higher than this IPCC default. While some increase could be expected due to partial degradation of readily degradable organics, the magnitude of this difference, along with the mismatch between sanitation types from which the default value comes from, underscores our central point that applying generalized influent-based conversion factors (e.g., COD:BOD = 2.4) to NSS containments can introduce significant uncertainty. The considerable disconnect between theory and reality underpins the urgency for *in situ* measurements of CH_4_ emissions from NSS containments to improve GHG estimates.

As stated previously, statistical patterns between measured parameters and CH_4_ emissions vary greatly among locations. For instance, while we found no correlation between measured CH_4_ emissions and *in situ* organic matter concentrations, a previous study observed a positive correlation between CH_4_ emissions and *in situ* COD and BOD mass in blackwater containments in Vietnam.^24^ Although in this study we evaluated cumulative CH_4_ production and, in the Vietnam study, they used normalized emissions, a conceptual rather than direct comparison is valid. The findings in Vietnam are counterintuitive under a steady-state framework (Figure 2), where it would rather be the difference between influent BOD and *in situ* BOD that is converted to CH_4_. Small differences in COD, BOD, and suspended solids between the influent and *in situ* accumulations were also observed.^24^ These smaller differences, combined with higher *in situ* concentrations, should actually result in lower CH_4_ production. Similarly to our study, another study in Vietnam found that CH_4_ emissions were more strongly influenced by factors such as the depth of the settled solids in containment and the time since last emptying than by BOD removal efficiency.^25^ Additionally, a study on septic tanks receiving both black and gray water in the USA found no correlation with measured *in situ* COD concentrations in either the first or second compartments.^18^ Overall, these findings highlight that the interpretation of organic loadings and concentrations strongly depends on sampling location, particularly since few studies measure BOD_0_ or changes in *in situ* BOD over time. This gap means that *in situ* measurements may not reflect actual variations in the degradation of organic matter, underscoring the limitations of assuming steady-state conditions during highly variable storage conditions. It could be plausible that the parameters in this study that did show correlations may better reflect these transitions in the internal conditions over the usage period.

#### 3.2.2 Reassessing Assumptions about Anaerobic Conditions and MCF Values

As presented above, net cumulative CH_4_ measurements were higher in Southern Coastal BC than in Kampala, despite cooler median *in situ* wastewater temperatures (15 °C vs 24 °C), higher DO concentrations (DO, 0.8 mg/L vs 0.1 mg/L), and less reducing conditions (ORP of −216 mV vs −280 mV) (Table 3). Based on the criteria of ORP between −200 and −500 mV, DO < 1.0 mg/L, pH between 5 and 8, and free ammonia levels below 1,500 mg/L,^15,17,59^ we defined all containments in this study as anaerobic. The degree of anaerobic activity indicating potential values for MCFs does not consistently align with current assumptions used by the IPCC, and similar discrepancies have been reported in the literature. For example, Diaz-Valbuena et al., found no relationship between temperature (ranging from 6–25 °C) and CH_4_ emissions, and no significant associations between CH_4_ fluxes and pH or ORP.^18^ Knappe et al., also found no correlation between CH_4_ flux and temperature.^23^ Conversely, Huynh et al., found CH_4_ emission rates correlated with more negative ORP, but not with temperature or lower DO.^24^ Although it is possible that responses to more extreme fluctuations in temperature could exist over different seasons or climatic regions not included in this study, in Southern Coastal BC the sampling period effectively captured the typical range of seasonal temperature variation, and in Kampala, the equatorial climate remains relatively stable year-round (Supplementary Methods S3). The organic matter in nonsewered wastewater is heterogeneous, and under storage conditions with little to no mixing, only a fraction is amenable to anaerobic degradation.^60,61^ Given that NSS containments are highly variable and complex ecological systems, it is not surprising that observed biological CH_4_ production does not agree with the current IPCC methodology, including MCF values. Overall, our results corroborate the literature and advocate for a critical reassessment of fundamental assumptions about MCF values in NSS containments.

### 3.3 Scaling-Up Measurements to Estimate CH_4_ Emissions

#### 3.3.1 Accounting for User Mobility and Containment Characteristics

Median per capita CH_4_ emissions, calculated using the IPCC assumption that the number of household residents is equal to PEs, were 30.8 g CH_4_ capita^–1^ day^–1^ (range of 0.2–153.2 g CH_4_ capita^–1^ day^–1^) in Southern Coastal BC and 12.6 g CH_4_ capita^–1^ day^–1^ (range of 0.7–41.5 g CH_4_ capita^–1^ day^–1^) in Kampala (Figure 3). These median values were on par with previously reported values using the same PE normalization approach (0.3–26.8 g CH_4_ capita^–1^ day^–1^).^18,20,23–26^ The IPCC default values (18 and 11 g CH_4_ capita^–1^ day^–1^ for Canada and Uganda, respectively^14^) fall within the range of our results, despite our central tendency values being higher. However, with PE estimates adjusted to 0.7 PE in Southern Coastal BC and 0.3 PE in Kampala, as explained in Table 1, median estimates increased to 43.9 g CH_4_ capita^–1^ day^–1^ (range of 0.2–218.9 g CH_4_ capita^–1^ day^–1^) and 41.9 g CH_4_ capita^–1^ day^–1^ (range of 2.3–138.3 g CH_4_ capita^–1^ day^–1^). The use of PEs has long been established for the planning and design of sewer-based, centralized WWTPs, and as people move through, into and out of a sewer-shed and use different toilets, the differences in loadings are averaged out.^59,62^ However, this concept does not translate to individual toilets, and there is currently no method to accurately reflect PEs based on individual toilet usage. In this study, literature-based estimates for total time spent at home (∼70% of time spent at home in HIC,^37^ and ∼30% in LMICs^38–40^ ) were used to hypothetically demonstrate the significant impact on reported emissions, rather than to recommend fixed values or an approach for all cases. Given the diverse patterns of toilet use, it remains unclear which PE assumptions most accurately reflect real-world daily loadings. Methods such as participant interviews could help further improve PE accuracy.^63^ Without a standardized way to define PEs, both absolute emissions and cross-regional comparisons are vulnerable to distortion. Until such data are available, caution is warranted in applying these specific PE values to policy or inventory calculations.

**Figure 3:**
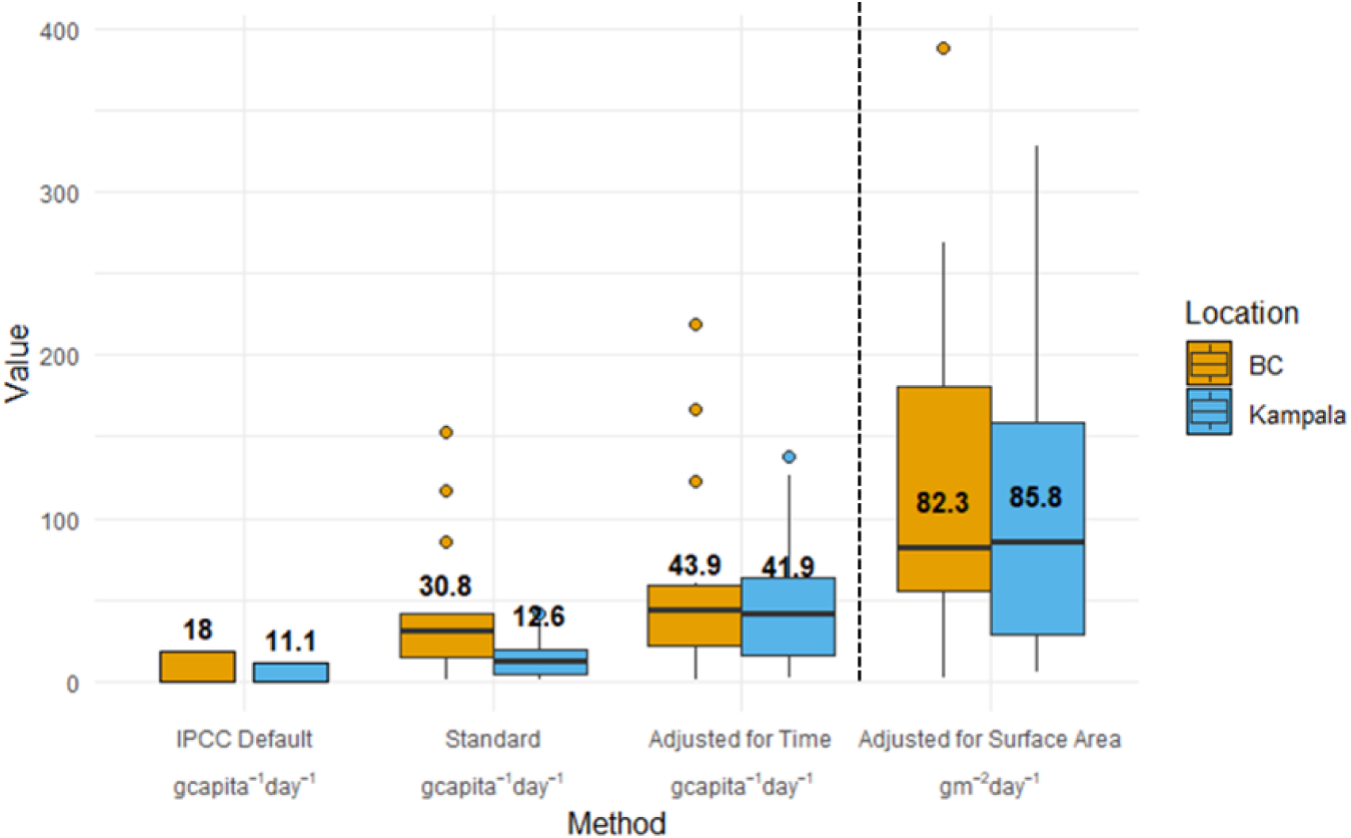
Comparison of median household CH_4_ emissions between Kampala and Southern Coastal BC across three normalization methods to IPCC defaults. Left: per capita, calculated as 1 user = 1 population equivalent (PE). Middle: per capita, adjusted for estimated time spent at home. Right: normalized by containment surface area. Boxes represent interquartile ranges (IQRs), lines and values indicate medians, and dots represent outliers.

Similarly to the adjusted per capita emissions, and in contrast to the standard per capita emissions, when CH_4_ measurements were normalized by surface area, median values were very similar in magnitude, with 82.3 g CH_4_ m^–2^ day^–1^ (range of 2.1–388.4 g CH_4_ m^–2^ day^–1^) in Southern Coastal BC and 85.8 g CH_4_ m^–2^ day^–1^ (range of 5.2–327.9 g CH_4_ m^–2^ day^–1^) in Kampala (Figure 3). This approach is well established in emissions reporting for wetland soil-based systems,^64^ leach fields,^65^ and landfills.^66^ Surface area emissions from wastewater in septic tanks and holding tanks could also be more robust when evaluating relationships with CH_4_ emissions, especially if PE assumptions are flawed or not standardized. NSS city-wide emission estimates could be built up by surface area of wastewater in septic tanks and holding tanks, and then normalized to PEs in a fashion similar to sewer-shed approaches.

Notably, Kampala’s median per capita estimate is more closely aligned with the IPCC Tier 1 default than Southern Coastal BC. Compared to each other, estimates were three times higher in Southern Coastal BC than in Kampala with the approach where one resident is equal to one PE. However, there were very similar values between locations with both the time-adjusted PE and surface area calculations. This sensitivity analysis was not intended to explain causation, but rather to show, how in the future, more informed time-adjusted PE values could impact estimated emissions per individual toilet. The apparent mismatch between measured fluxes and simplified theoretical expectations (i.e., IPCC defaults) underscores why current scaling approaches may not adequately capture the complexity of NSS containment emissions, as they are difficult to reconcile with field observations. For example, when we compared our non-normalized cumulative CH_4_ concentrations, they showed much less variation in relative magnitude between individual containments as well as between Southern Coastal BC and Kampala—differences that were, in fact, not statistically significant. Importantly, what this outcome does demonstrate is that correlations with questionnaire and physicochemical parameters—such as time since last emptied, wastewater depth, scum depth, temperature, DO, and ORP concentrations—could change dramatically with different normalization methods, which is why we chose to report and use our unscaled values in our interpretation of potential predictors.

#### 3.3.2 Reevaluating MCF Estimation Approaches for NSS Containments

As shown in Figure 2, BOD_0_ values are an important component of the current IPCC method, which are estimated per household resident and, as our results indicate, require improvement. To demonstrate this, we used the IPCC default BOD per capita loading values (i.e., 60 g BOD capita^–1^ day^–1^ for Canada and 37 g BOD capita^–1^ day^–1^ for the continent of Africa^14^) to calculate MCF values, as this is what has been done in existing literature to date.^18,20,24,25^ Combining these with our field-measured CH_4_ emissions yielded MCF values of 0.57–0.86 (Table 5). These MCF values exceed both the IPCC default of 0.5 and the range we calculated from other field studies (<0.1–0.5), even though the measured median CH_4_ emissions (12.6–30.8 g CH_4_ capita^–1^ day^–1^) in this study were broadly consistent with the literature (11–25.5 g CH_4_ capita^–1^ day^–1^ globally) (Table 5), due to the range of regional default BOD loading values given in the IPCC.

**Table 5:** Comparison of Measured Methane Emissions and Calculated Methane Correction Factors in This Study and Literature. Values Marked with † are from Measurements Taken Directly in the Field and Values Marked with * are Calculated Using BOD Loading Values from the IPCC Tier 1 Methodology^a^,^b^,^c^,^d^

				Methodological Approach				
Author & Year	Area	Establishment Category	Type and Study Size	BOD_0_	PE	Gas	Methane Emissions (g CH_4_ capita^–1^day^–1^)^a^	Methane Correction Factor (MCF)^d^
This study	Southern Coastal BC, Canada	Household Septic Tank	Single Family, *n* = 16	Canada (60 g capita^–1^ day^–1^)	IPCC + Adjusted	Floating Flux Chamber	30.8**†** (0.2–153.2)	0.86*
						**Median Values**		
This study	Kampala, Uganda	Household Holding Tank	Multi-Family, *n* = 17	Uganda (37 g capita^–1^ day^–1^)	IPCC + Adjusted	Floating Flux Chamber	12.6**†** (0.7–41.5)	0.57*
						**Median Values**		
Diaz-Valbuena et al. (2011)^18^	California, USA	Household Septic Tank	Single Family, *n* = 8	USA (85 g capita^–1^ day^–1^)	IPCC	Modified Flux Chamber	11 (±2)**†**	0.22*
						**Geometric Mean**		
Truhlar et al. (2016)^20^	New York, USA	Household Septic Tank	Single Family, *n* = 8	USA (85 g capita^–1^ day^–1^)	IPCC	Vent Gas Flux	11 (±12)**†**	0.22*
						**Mean**		
Huynh et al. (2021)^24^	Hanoi, Vietnam	Household Septic Tank	Single Family, *n* = 10	Asia (40 g capita^–1^ day^–1^)	IPCC	Floating Flux Chamber	11.9 (±4.9)**†**	0.5*
						**Mean**		
Moonkawin et al. (2023)^25^	Hanoi, Vietnam	Household Septic Tank	Single Family, *n* = 23	7–11 g capita^–1^ day^–1^ (measured)	IPCC	Floating Flux Chamber	10.9 (range: 2.2–26.8)**†**	0.45*
						**Mean (After IQR Filtering)**		
Knappe et al. (2022)^23^	Limerick, Ireland	Household Septic Tank	Single Family, *n* = 2	Europe (60 g capita^–1^ day^–1^)	IPCC	Modified Flux Chamber	0.3^b^ **†**	0.008*
						**Median**		
Ross et al. (2020)^26^	Rhode Island, USA	Household Septic Tank	Single Family, *n* = 17	USA (85 g capita^–1^ day^–1^)	IPCC	Closed Chamber connected to Picarro© Gas Analyzer	6.3^c^ **†**	0.12*
						**Median**		
IPCC Defaults^14^	Global	Septic Tank	N/A	37–85 g capita^–1^ day^–1^	IPCC	IPCC Guidelines	25.5* (USA) 18.0*	0.5* (range: 0.4–0.72)
							(Canada) 11.1* (Uganda)	

a IPCC approach (1 resident = 1 person equivalent). Ranges and standard deviations were reported where possible.

b Based on the reported value of 2.72 kg-CO_2_ eq capita^–1^ year^–1^and the 100-year GWP of 27.9.^12^

c Based on the reported value of 176 g CO_2_eq capita^–1^ day^–1^and the 100-year GWP of 27.9.^12^

d Field emission rates (median or mean) are normalized using BOD loading rates (kg BOD capita^–1^ day^–1^) from Table 6.4 of the 2019 IPCC Refinement (USA: 0.085, Europe/Canada: 0.060, Africa: 0.037, Asia = 0.04) and maximum methane production (0.6 kg CH_4_/ kg BOD).^14^

The IPCC default per-person BOD loadings range from 37 to 85 g BOD capita^–1^ day^–1^ ^14^ and have notable limitations. For example, in North America, the USA and Canada are assigned regional averages of 85 and 60 g BOD capita^–1^ day^–1^, respectively, while the entire African and Asian regions are each represented by a single average value of 37 and 40 g BOD capita^–1^ day^–1^, respectively. This is problematic, as these values are a great oversimplification of the diverse contexts in these regions and because they are not based on direct measurements of domestic wastewater entering containments. Instead, these values are derived from wastewater data collected in the USA and extrapolated globally using assumptions that regional income level and diet predict BOD loadings.^56^ While it has been claimed that differences in diet can explain regional differences in wastewater,^67^ existing studies provide limited evidence^7,68^ or rely on inappropriate constructs, such as race as a biological determinant.^69^ Measured values for BOD_0_ are sparse. One review reported BOD loadings between 14 and 33.5 g BOD capita^–1^ day^–1^ for excreta only,^67^ while the only study to measure both BOD loadings and GHG emissions in NSS reported an average of 7 to 11 g BOD capita^–1^ day^–1^ from blackwater septic tanks in Vietnam.^25^ In contrast, a study in Indonesia reported an average of 175.5 g BOD capita^–1^ day^–1^ for a household septic tank.^70^ Notably, although the study in Vietnam did measure BOD_0_, the measured values were not used to calculate an MCF. If the BOD_0_ values from the study in Indonesia or Vietnam were applied instead of the IPCC default value for Asia (40 g BOD capita^–1^ day^–1^) the resulting MCFs would differ substantially (2.6 and 0.1, respectively). An MCF greater than one is physically implausible and highlights how sensitive the calculation is to the assumed input loading. Consequently, this further highlights the research need to understand loadings of individual NSS containments or apply concepts that are less sensitive to individual-level fluctuations, such as surface area emission values normalized regionally.

#### 3.3.3 Methodological Variability

Methods to generate CH_4_ estimations lead to inconsistencies via two main channels. First, sampling and measurement methods are not standardized, with a range of techniques, including floating flux chambers, vent gas collection, onsite analyzers (e.g., Picarro ©), and off-site gas chromatography (Table 5), potentially influencing reported emissions. While floating flux chambers are among the most commonly used tools, their accuracy depends heavily on design specifics, sealing integrity, and the depth of insertion in liquid layers.^71^ Variations in fan use and external power requirements can also introduce leak points or limit applicability in resource-constrained settings.^71^ In addition, while each technique has its own strengths and limitations, most studies do not report calibration procedures or evaluate the performance of their equipment under field conditions. As with the challenges discussed around PEs, additional variations in reporting metrics (e.g., mean of non-normal data vs median), sampling scales, and measurement techniques make it difficult to determine whether observed differences in emissions reflect true variability in degradation dynamics or are simply artifacts of study design. Second, inappropriate summary statistics impede the comparison and interpretation of emission values across the literature. The data analyzed in this study were non-normally distributed. As such, the median was selected as the most representative measure of central tendency, supported by a sensitivity analysis comparing it to alternative statistics (Figures S129–S130). This approach mitigates the influence of extreme outliers, which are common in highly variable systems like NSS.^35^ Only two other studies in the literature accounted for this: (i) by using a geometric mean^18^ and (ii) applying interquartile range (IQR) filtering prior to statistical analysis.^25^ This absence of standardization is especially problematic for highly variable systems like NSS, where methodological consistency is critical to ensuring comparability, building robust emission factors, and supporting the scaling up of direct point-source measurements to the city or regional level.

## 4 Implications

This study highlights that two current assumptions used in the IPCC methodology do not reflect the reality of NSS containment complexity and variability. Specifically: (1) the assumptions around *in situ* degradation that MCFs are based on, and (2) the implications of using PEs to scale emissions and their ability to represent influent BOD loading. This work identifies critical knowledge gaps to improve NSS containment GHG emission estimates from point sources to regional scales, with good practice recommendations and implications for policy.

In this study, similar to other recent scientific studies,^18,20,23–26^ we evaluated relationships between CH_4_ emissions and *in situ* properties of wastewater in storage, but no relationships were observed (Table 4). This highlights the need for a mechanistic understanding of factors impacting biological CH_4_ production during storage in containments, as they are currently not understood, and would inform assumptions that IPCC MCFs are based on (i.e., why MCFs require an “improved understanding” in Figure 2). In this study, which considered septic tanks in Southern Coastal BC and holding tanks in Kampala, scum layers were observed, but statistically significant increases in settleable solids did not occur near the bottom of the containments. Additionally, no statistically significant stratification based on any physicochemical characteristics was detected between the three sampled depths. In Zambia, differences in microbial communities in both holding tanks and pits have been linked to water usage, wastewater properties, and metrics of stabilization, as well as downstream treatment performance such as dewatering.^72^ This suggests that the influence of community-specific metabolic traits, such as sulfur reduction, methanogenesis, and ammonia tolerance, should be considered to fully understand MCF values, so they can accurately reflect real conditions. Few studies have evaluated seasonal and diurnal variations in biological CH_4_ production,^23,26^ and most studies of CH_4_ dynamics in containments assume steady-state conditions. Understanding microbial community structure and function, and the impacts that seasonal and environmental factors have, is central to moving beyond correlation and toward causal mechanisms.

If there is a continued reliance on individual point-source measurements as a method of data collection, key knowledge gaps need to be filled, along with standardized, replicable, and inexpensive approaches to measure and scale these measurements. This study found that the only relationships between point-source *in situ* CH_4_ emissions were observed with field measurements that included the number of users, wastewater depth, time since last emptied, and scum depth, which has a two-fold implication. First, it means that further research is needed to improve our understanding of PEs and BOD_0_ loadings per PE, as there was a lack of correlation between *in situ* physicochemical characteristics and CH_4_ emissions (i.e., why PE requires an “improved understanding” in Figure 2). Although influent BOD_0_ values to containments are extremely difficult to quantify, there remains a need to define the relationship between what is being loaded into these containments and what accumulates *in situ*, as this amount of change in organic matter is what is being converted to CH_4_. Second, a better understanding of any relationships to readily collectible, cost-effective demographic, environmental, and technical data (e.g., number of users, wastewater depth, scum depth, time since last emptied) needs to be explored for scaling up to city-wide estimates.^10^ Alternatively, another promising approach for city-wide estimations of CH_4_ emissions is to rely on surface area flux (Figure 3), which could then be divided by the total population to reduce the sensitivity to the high variability based on PEs and BOD_0_ loadings at the individual containment level. GHG estimates should include transparently reported primary emissions data, methodologies, and underlying assumptions to support consistent interpretation across studies. This study has highlighted how GHG estimates can vary depending on how point-source measurements are scaled up. Further collaboration across the sector is needed to develop and adopt standard methods for data collection and analysis, reducing discrepancies in reported emissions.

Researchers and practitioners should also incorporate dissolved CH_4_ in field study design and interpretation. Although some studies that have measured dissolved CH_4_ have concluded there is negligible contribution (e.g., dissolved CH_4_ in *in situ* wastewater as 0–11%,^18^ and 0.04%^24^ of the total CH_4_ emitted from flux chamber measurements), it will likely be emitted as wastewater is mixed during emptying or at delivery to treatment. For example, a recent study quantifying CH_4_ released during transport in sewers to centralized WWTPs estimated that this source of dissolved CH_4_ could reach up to 78 g CH_4_ capita^–1^ year^–1^.^73^ Given the relatively low levels of degradation of organic matter that occur during storage,^7,25^ biodegradable organic matter that will be released downstream in the sanitation service chain also needs to be taken into account, as it is likely a non-negligible fraction of CH_4_ emissions that are unaccounted for in current inventories.

CH_4_ emissions from NSS containments are neither negligible nor uniform, as demonstrated by this study. They have been estimated to account for roughly 0.2% of global anthropogenic carbon emissions.^3^ While these emissions are important to consider, we must also acknowledge the critical role this sanitation infrastructure plays in closing essential sanitation gaps, as well as addressing sanitation’s fundamental aims of public health, dignity, and well-being as a basic human right.^74^ Despite substantial uncertainties in NSS GHG emissions, for the time being, researchers, practitioners, and policymakers will have to rely on the best available evidence to make informed decisions with limited information. Within an iterative framework, these findings aim to support continued methodological refinement and guide the collection of more comprehensive and informative data in the future.

## Supplementary Material



## Notes

Background and collected data; additional results; summary of sampling parameters, methods, and quality control and assurance protocols; description of field sampling equipment; additional results of physicochemical analysis; mass-flux calculation methodology and results; gas concentration measurement methodology; copy of the administered questionnaire; additional discussion; supplementary references; access to Eawag Research Data Institutional Collection (ERIC) repository: 10.25678/000FPN­(PDF)
