# Supplementary material for: Improving Methane Emission Estimates from Nonsewered Wastewater during Storage in Septic and Holding Tanks
Source: Environ Sci Technol. 2026 May 12;60(20):14467–81. doi: 10.1021/acs.est.5c17325 (PMC13217549; doi:10.1021/acs.est.5c17325)
Supplement: Supplementary file 1 [file es5c17325_si_001.pdf]

Supplementary Information: Improving Methane Emission Estimates from Non-Sewered Wastewater During Storage in Septic Tanks and Holding Tanks

Authors

Kelsey Shaw[1,2], Charles B. Niwagaba[3], Linda Strande[2]*, and Caetano C. Dorea[1]*

[1]Department of Civil Engineering, University of Victoria, Victoria, British Columbia, V8P5C2, Canada

[2]Sandec: Department of Sanitation, Water and Solid Waste for Development, Eawag: Swiss Federal Institute of Aquatic Science and Technology, 8600 Dübendorf, Switzerland

[3]College of Engineering, Design, Art and Technology, Makerere University, P.O. Box 7062, Kampala, Uganda

***Corresponding Authors:** caetanodorea@uvic.ca & linda.strande@eawag.ch

Pages: 16

Tables: 21

Figures: 132

Table of Contents

Supplementary Methods S1 - Sampling Parameters, Methods & QA/QC 3
Supplementary Methods S2 - Field Sampling Equipment 5
Grab Sampler 5
Modified Floating Flux Chamber 5
Supplementary Methods S3 - Physicochemical Analysis 7
Mass Flux Calculation Methodology 7
Gas Concentration Measurements 9
Supplementary Methods S4 – Copy of Questionnaire 11

List of Tables

[*In html document*]

Table S1: Greenhouse Gas Dataset Variable Names, Data Types, and Descriptions
Table S2: Percentage of Missing Values by Parameter Across All Datasets
Table S3: Containment Characteristic Categorical Data from Questionnaires and Onsite Measurements
Table S4: Containment Characteristic Numeric Data from Questionnaires and Onsite Measurements (Household Establishments)
Table S5: Containment Characteristic Numeric Data from Questionnaires and Onsite Measurements (Commercial Establishments)
Table S6a: Statistical Testing Results for Numeric and Non-Numeric Containment Questionnaire and OnSite Data
Table S6b: Testing regional differences across scaled numeric parameters
Table S6c: PERMANOVA on Euclidean distances of scaled numeric parameters
Table S6d: Homogeneity of multivariate dispersion by country
Table S7: Wastewater Data for each Containment by Sampling Depth (Top, Middle, Bottom)
Table S8: Wastewater Data for Each Containment Averaged across Sampling Depths
Table S9: Statistical Testing Results for *In Situ* and Laboratory Wastewater Data by Region
Table S10: Statistical Testing Results for *In Situ* and Laboratory Wastewater Data by Establishment Type
Table S11: Statistical Testing Results for *In Situ* and Laboratory Wastewater Data by Sampling Depth (Top, Middle, Bottom)
Table S12: Spearman's Rank Correlation Analysis for Household Containment and Wastewater Data by Region
Table S13: Summary of Fitted *In Situ* Methane Data Slopes and R^2 Values for Each Containment
Table S14: Cumulative Concentrations (ppm) of GHGs for Each Sampled Containment
Table S15: Summary Table for Methane Emissions with Different Population Equivalent Options (g/cap-day) and per Surface Area (g/m^2-day)
Table S16: Methane Correction Factor (MCF) Calculations Using Median Emission Rates for All Population Equivalent Options

Table S17: Summary of Median Cumulative CH_4 Emissions by Region and Establishment Type
Table S18: Summary of all Spearman Rank Correlation Results for Southern Coastal BC and Kampala between Questionnaire, *In situ* and Lab Data and Cumulative Methane Emissions

[*In SI Document*]
Table S19: *In situ* Sampling and Survey Quality Assurance and Control (QA/QC)
Table S20: Laboratory Parameters Quality Assurance and Control (QA/QC)
Table S21. Summary of Fitted CH_4 Slopes and R^2 Values for Each Household Containment

List of Figures

[*In html document*]
Figure S1: Parameters with Missing Values Above 5%
Figures S2-S28: Distribution of 'non-numeric questionnaire data' by region
Figures S29-S37: Boxplots of 'numeric' questionnaire data by Region and Establishment Type
Figures S38-S60: Boxplots of 'physico-chemical + biological data' averaged across sampling depths - by region and establishment type
Figures S61 - S83: Boxplots of physico-chemical + biological data - Unaveraged across sampling depths - by region and establishment type
Figure S84: Strong + Significant Spearman Rank Correlations in Southern Coastal BC Households
Figure S85: Strong + Significant Spearman Rank Correlations in Kampala Households
Figure S86: Overlap of Strong and Significant Spearman Rank Correlations by Region
Figure S87-S125: Cumulative Methane vs Time for each containment sampled.
Figure S126: Relationship between Cumulative CH^4 vs CO^2 Emissions
Figure S127: Household Population Equivalent Standard Method (Option 1) and Time-Spent-at-Home Adjusted Emission Rates (Option 2) for Each Region
Figure S128: Surface Area Emission Rates for Each Region
Figure S129: Comparison of different central tendency calculations for each region and establishment type for Population Equivalent Emissions Option 1
Figure S130: Comparison of different central tendency calculations for each region and establishment type for Population Equivalent Emissions Option 2

[*In SI Document*]
Figure S131. Multi-Stage Wastewater Grab Sampler
Figure S132. Modified Floating Flux Chamber Design for Use on Liquid Surfaces

Supplementary Methods S1 - Sampling Parameters, Methods & QA/QC

For more details please refer to the Swiss Federal Institute for Aquatic Science and Technology (Eawag) research data institutional repository (ERIC) https://doi.org/10.25678/000FPN and https://supplementary-material-shaw-ghg.netlify.app/ which contains additional tables and figures.

Table S19. *In situ* Sampling and Survey Quality Assurance and Control (QA/QC)

Parameter	Equipment / Method	QA/QC
Dissolved Oxygen (DO)	Microx 4: with 5 meter DO dipping probe and temperature probe (Presens, Germany).	As per manufacturers instructions x1 a week when in use.
Temperature		
pH	HQ4300 Portable Multi Meter (HACH).<br>pH rugged 5 meter probe.	Standard commercially available calibration solution from HACH, x1 a week when in use.
Oxidation Reduction Potential (ORP)	HQ4300 Portable Multi Meter (HACH).<br>ORP rugged 5 meter probe.	
Electrical Conductivity (EC)	HQ4300 Portable Multi Meter (HACH).<br>EC rugged 5 meter probe	
Depth	Collapsible avalanche probe (Commercially available; length of 0-5 meters).	N/A
Surface Area & Volume	Volaser measuring device (refer to users guide[1]).	As per user manual recommendations, measurements taken until containment shape confidence achieved.
Questionnaire Data	Refer to Supplemental Methods S4 – Questionnaire.	Translated and administered in Luganda in Kampala (Uganda).

Table S20. Laboratory Parameters Quality Assurance and Control (QA/QC)

Parameter	Method	Replicates	QA/QC
Chemical Oxygen Demand (COD)	Method 5220D of Standard Methods [2], with test kits as per manufacturers directions (HACH: TNT823/825 or LCK014/114).	Every 6th sample analyzed in triplicate (≥10% of total samples)	Triplicates within 10%, accepted if under 20% of average.
Soluble COD (sCOD)			
Biochemical Oxygen Demand (BOD)	Method 5210B of Standard Methods [2]. DO Dipping Probe (see **Table S19**).	Every 6th sample analyzed in triplicate (≥10% of total samples)	Triplicates within 10%, accepted if under 20% of average.
Total Organic Carbon (TOC)	DIN EN ISO 20236<br>Catalytic combustion 720°C --> measurement of CO_2 using NDIR.	Every 6th sample analyzed in triplicate (≥10% of total samples)	As specified in Standard method / ISO Norm.
Total Solids (TS)	Method 2540B of Standard Methods [2] as modified in [3].	Every 6th sample analyzed in triplicate (≥10% of total samples)	Triplicates within 5% of average.
Total Suspended Solids (TSS)	Method 2540D of Standard Methods [2] as modified in [3].		
Volatile Solids (VS)	Method 2540E of Standard Methods [2] as modified in [3].		
Volatile Suspended Solids (VSS)	Method 2540D and E of Standard Methods [2] as modified in [3].		
Silica Content	Method DWA-M 383 for the determination of sand content (DWA, 2008) as modified in [3].		
Total Nitrogen (TN)	Method 4500-N C of Standard Methods [2], with test kits as per manufacturers directions (HACH: TNT827 or LCK238).	Every 6th sample analyzed in triplicate (≥10% of total samples)	Triplicates within 10%, accepted if under 20% of average.
Ammonium (NH_4-N)	Method 4500 of Standard Methods [2], with test kits as per manufacturers directions (HACH: TNT832 or LCK302).		
Nitrite (NO_2-N)	Method 4500-NO2-B of Standard Methods [2], with test kits as per manufacturers directions (HACH: TNT839 or LCK341).		
Nitrate (NO_3-N)	Method 4500-NO3-E of Standard Methods [2], with test kits as per manufacturers directions (HACH: TNT836 or LCK340).		
Total Phosphorus (TP)	Method 4500-P of Standard Methods [2], with test kits as per manufacturers directions (HACH: TNT845 or LCK350).		
Orthophosphate (PO_4-P)			
Sulphide (S)	Method 4500-S of Standard Methods [2], with test kits as per manufacturers directions (HACH: TNT861 or LCK653).		

Supplementary Methods S2 - Field Sampling Equipment

Grab Sampler

A custom grab sampler, inspired by another study conducted in Kampala Uganda [4], was designed and fabricated at the Eawag workshop (Dübendorf, Switzerland) using locally available materials. The sampler was specifically designed to collect wastewater samples from various depths within containments while minimizing cross-contamination from wastewater in other layers (**Figure S131**). The device functioned by allowing the sampling container to open and close at precise depths, ensuring accurate sample collection. Once retrieved, the collected samples were transferred into 1L polyethylene Nalgene bottles for secure transport to the lab for further analysis.

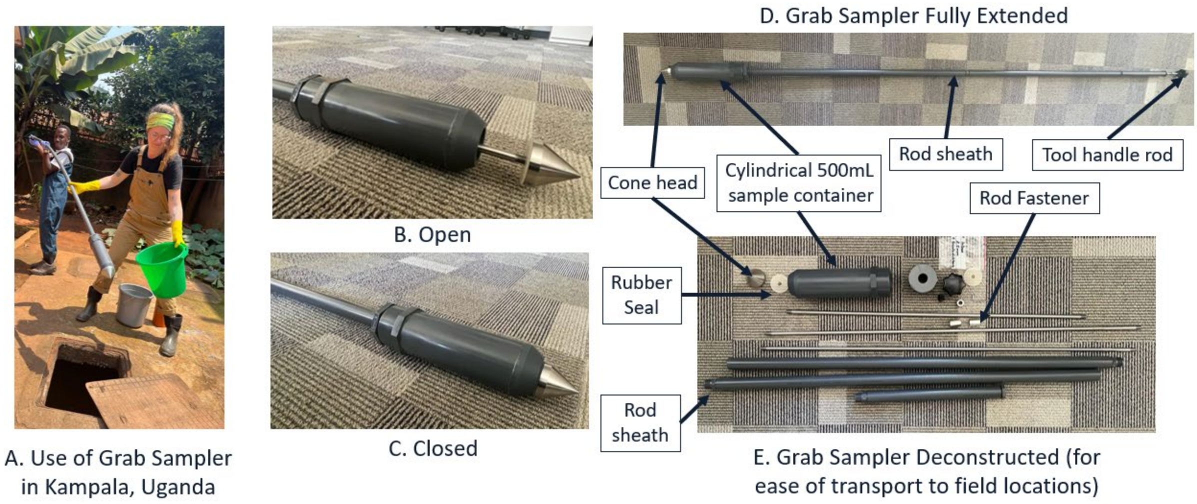

Figure S131. Multi-Stage Wastewater Grab Sampler

Modified Floating Flux Chamber

The chamber was constructed from a 4-inch diameter, 12-inch tall polyvinyl chloride (PVC) pipe with a sealed cap, as illustrated in **Figure S132** and based on the design principles outlined by a similar study in the United States [5]. A small vent tube was incorporated to prevent pressure buildup from gas emissions. The chamber was suspended from a tripod above the containment opening, allowing it to float freely. To maintain an airtight seal, the chamber was submerged a minimum of 2 cm underwater, with precise measurements taken each time it was deployed. Importantly, the surface scum was left undisturbed, following the methodology of Huynh et al. (2021) [6]. This approach assumed that gas would naturally penetrate through the surface layer while minimizing any disruption to the existing

conditions. This method was chosen to preserve *in situ* conditions and avoid altering the gas diffusion pathway or chamber seal integrity.

A fan was not incorporated into our flux chamber design due to methodological concerns regarding additional fittings and penetrations in the chamber that would increase potential leak pathways and add mechanical complexity in field deployments [7]. This consideration is particularly relevant in rural or resource-constrained settings, like those in which we sampled, where robustness and simplicity are critical. To be able to ensure samples were well-mixed and reproducible the flux chamber was designed to have a small headspace volume (3.3 L, typically ≤2 L when submerged), such that molecular diffusion was expected to rapidly equilibrate gas concentrations during short deployments [8]. To further ensure representative headspace sampling, a waste volume was withdrawn using the syringe prior to the time-zero sample to clear stagnant gas from the sampling port, and the syringe was pumped several times through the airtight port to promote additional mixing. Chambers were leak-tested prior to deployment and validated under controlled laboratory conditions using standard gases of known CH_4 and CO_2 concentrations to confirm reproducibility and analytical accuracy.

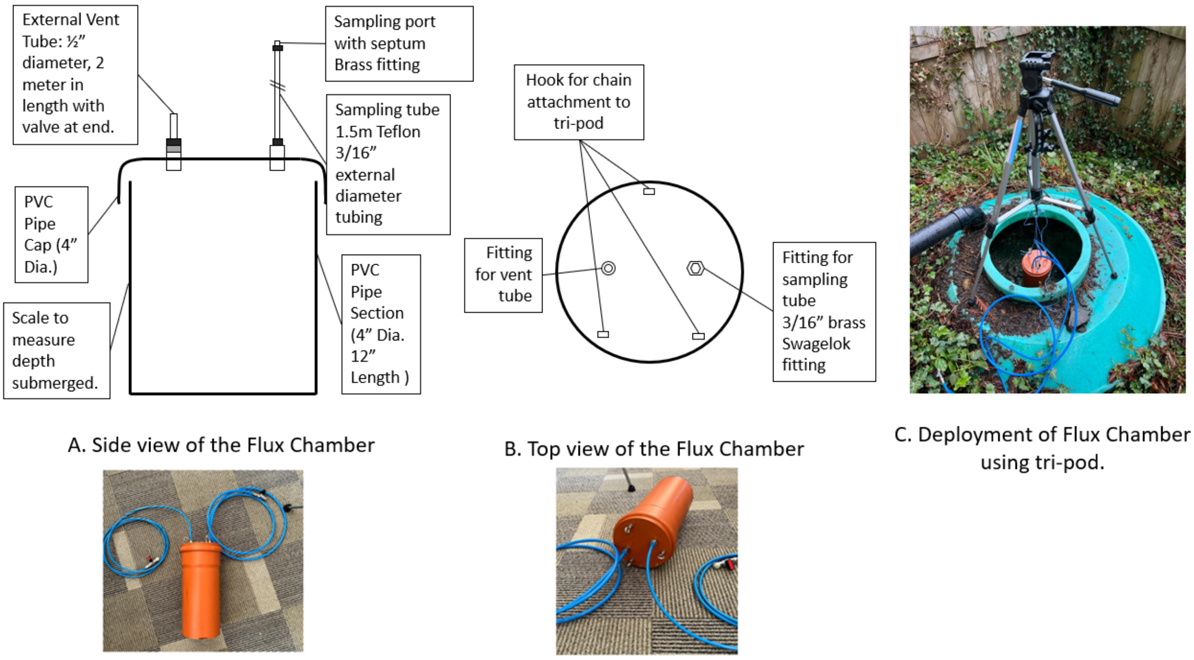

Figure S132. Modified Floating Flux Chamber Design for Use on Liquid Surfaces

Supplementary Methods S3 - Physicochemical Analysis

For more details please refer to (https://supplementary-material-shaw-ghg.netlify.app/) which contains additional tables and figures.

Although capturing seasonal variation was not an objective of this study, ambient air temperatures were recorded on sampling days as well as *in situ* temperatures at the top, middle, and bottom of each containment (**Figure S62**) where no statistically significant differences were found (**Table S11**). In Southern Coastal BC, the sampling period effectively captured the typical range of seasonal temperature variation (**Figure S39**). It should also be noted that during method and instrument testing conducted in December of 2022, wastewater temperatures remained consistent with those recorded during spring sampling in this study in April-May of 2023. This is due in part because the containments are buried and receive temperate influent, which buffers against short-term atmospheric changes. In Kampala, the equatorial climate is relatively stable year-round.

Mass Flux Calculation Methodology

The closed flux chamber principal method was used to calculate gas emissions [9]. The flux chamber was lowered into the containment. Gas samples were collected at regular intervals over a 30 minute period at 0, 5, 10, 20 and 30 minute time steps. This method relies on using the headspace volume and concentration/time to determine the emissions rate. As this method does not require the flow rate to be measured, it is ideal for when flow rate values are low [7].

All gas concentration measurements were converted to mg/m^3 using the following formula:

$$C_{mg/m3} = \frac{C_{ppm} 10^6 \times MW \ \times 1000}{RT/_{P}}$$

Where C_{ppm} is the concentration of gas in ppm, as determined by gas chromatography (GC). *MW* is the molecular weight of the gas under consideration (g/mol), *R* is the ideal gas constant (0.000082057 atm-m3/mol-K), *T* is the absolute temperature (K), *P* is the absolute pressure of the gas (atm).

The linear plot method was used, a summary table of the slopes derived from this linear fit and corresponding R^2 values are presented in **Table S21,** the corresponding figures and full data set are available in the html document (**Figures S87-S125**).

Table S21. Summary of Fitted CH_4 Slopes and R^2 Values for Each Household Containment

Southern Coastal BC			Kampala		
**ID**	**Slope**	**R^2**	**ID**	**Slope**	**R^2**
1	145.58	0.99	1	50.23	0.99
2	201.22	0.97	2	52.67	0.99
3	31.21	1.00	3	139.82	0.93
4	605.04	0.98	4	314.30	0.99
5	774.10	0.97	5	525.12	0.99
6	247.19	0.98	6	256.26	0.99
7	357.36	0.98	7	17.26	0.87
8	9.71	0.89	8	20.48	0.99
9	903.41	0.95	9	147.86	1.00
10	1244.51	0.99	10	432.87	0.99
11	1685.72	1.00	11	831.26	0.96
12	263.69	0.93	12	545.76	0.98
13	232.11	0.81	13	297.86	0.99
14	1052.24	0.97	14	623.22	1.00
15	406.28	0.96	15	754.88	0.98
16	375.81	1.00	16	1035.17	0.97
			17	99.89	0.98

The slope, m, was used to compute the flux using the following equation:

$$E_{cap} = m \times 1440 \times 1000 \times V_{FC} \times \frac{A_{con}}{A_{FC}} \times n$$

Where, *Ecap* is the emission rate per capita (g CH_4 capita^{-1} day^{-1}), *m* is the gradient of the linear regression, 1440 is minutes in a day (min/ day), V_{FC} is the volume of the FC (m^3), A_{con} is the surface area of the containment (m^2), A_{FC} is the surface area of the FC (m_2), and *n* is the number of people using the containment.

The emission rate of gas (E_{con}) from the whole containment was calculated as below:

$$E_{con} = EFC \times A_{con}$$

Where, E_{con} is the emissions rate of the whole containment (g CH_4 capita^{-1} day^{-1}), *EFC* is the emission rate from the flux chamber (g CH_4 capita^{-1} day^{-1}), and A_{con} is the surface area of the containment (m^2). This can easily be converted to per capita by dividing by the total number of people that use the containment, or a population equivalency number.

Southern Coastal BC containments are predominantly two-compartment precast tanks featuring a dividing wall that leaves an opening between the 1st and 2nd compartments, while Kampala containments were predominately single-chamber. To enable robust cross-site comparisons despite these configuration differences, and address potential variation due to flux chamber placement, we adopted the conservative "maximum emission scenario" from Huynh et al., (2021) [6] where fluxes were measured exclusively from the first compartment (or full single chamber, as was the case in Kampala) and were scaled by the total tank surface area. This approach assumes uniform per-area emission rates across compartments, yielding an upper-bound total that strengthens BC-Kampala emission comparisions. Our 'Volaser' measurement device facilitated precise total surface area measurements via a free-rotating laser that could scan across liquid surface and between chambers, ensuring methodological consistency [10].

Gas Concentration Measurements

For the analysis of CH_4, CO_2, and N_2O concentrations, we employed a GC method (2010 Shimadzu, Germany) using a Flame Ionization Detector (FID) and an Electron Capture Detector (ECD). CH_4 was analyzed directly with the FID, while CO_2 was first reduced to CH_4 before detection by the FID. N_2O was analyzed using the ECD. The GC setup featured a Carboxen 1010 PLOT column for CH_4 and CO_2 analysis and a GS-Carbonplot column for N_2O, with split-less injection at 200°C and detection at 350°C. The detection limit for CH_4 was below 1 ppm. The analysis was conducted at Eawag (Kastanienbaum, Switzerland).

Gas samples were stored and shipped at ambient temperature in vacuum-sealed 12 mL glass vials to prevent leakage and contamination. During fieldwork, sets of field control vials containing standard lab gas mixtures (70% CH_4) were transported and handled under identical conditions as the field samples, including exposure to the same temperature fluctuations and shipping durations. These field controls were analyzed concurrently with the samples upon arrival in the laboratory to verify the integrity of gas concentrations during transport and storage. All control samples returned the expected CH_4 concentrations (within 1% by volume), confirming that no significant changes occurred during sampling, storage, or transport. Field controls were prepared in triplicate for each batch of vials used in the field to ensure continued validity of the procedure.

Samples were collected in the 12 mL vials for easy transport and later diluted in 120 mL serum bottles with standard gas to ensure they fell within the optimal concentration range for analysis. During each chamber deployment, five gas samples were collected over the 30-minute measurement period at 0, 5, 10, 20, and 30 minutes. To assess analytical precision and reproducibility, duplicate samples were collected for at least 10% of all individual vials. Duplicate sampling was distributed across the dataset such that each of the five time points (0, 5, 10, 20, and 30 minutes) was represented by duplicates across the full set of containments sampled. All duplicate gas vials met QA/QC criteria (i.e., within 10% agreement) when analyzed on the GC-FID, confirming consistency of concentration measurements. The full analytical protocol is documented in the Eawag internal SOP (41-016-004), which details GC parameters and sample preparation in which similar published methods are available in a study by Khatun et al., (2024) [11].

Supplementary Methods S4 – Copy of Questionnaire

***In situ* assessment and collection of wastewater from non-sewered sanitation containments**

Adapted from 'Methods to Reliably Estimate Faecal Sludge Quantities and Qualities for the Design of Treatment Technologies and Management Solutions' [12].

Note on Eligibility Criteria for Convenience Sampling Campaign: All selected containments were deemed operational, accessible for sampling equipment, participant's expressed willingness through ethical consent process, and the setting was rural for Southern Coastal BC and urban for Kampala.

Location

1. What is the neighborhood/local area name?

____

Type of establishment

2. What type of establishment does the containment serve?
   - ☐ Household (single)
   - ☐ Household (multiple)
   - ☐ Other (specify): _______________________________________

User information

3. Describe toilet users
   - Is this toilet shared between multiple households?
     - ☐ Yes
     - ☐ No
   - What is the number of users? _______________________
   - Do people pay to use this toilet?
     - ☐ Yes
     - ☐ No
   - Do you rent or own your home? _______________________

Describe the onsite containment:

4. How would you classify this type of containment?
   - ☐ Pit latrine
   - ☐ Septic tank
   - ☐ Holding tank

5. Describe the containment's lining:
   - ☐ Fully lined (watertight)
   - ☐ Partially lined (water permeable)
   - ☐ Unlined
   - ☐ I don't know

- If fully or partially lined, what material is the lining made of?
  - ☐ Concrete
  - ☐ Fiberglass
  - ☐ PVC or plastic
  - ☐ Brick/stone/cinderblock
  - ☐ Wood
  - ☐ Other (specify):_________________________________

6. Do you notice a change in the liquid level inside the containment between rainy and dry seasons?

- ☐ Yes
- ☐ No
- ☐ I don’t know
- If Yes, what is the difference?
  - ☐ Liquid level is higher during the rainy season
  - ☐ Liquid level is lower during the rainy season
  - ☐ Other (specify): ____________________

7. Does the containment have one or more baffles?

- ☐ Yes
- ☐ No
- ☐ I don’t know

8. Does the system have an outflow?

- ☐ Yes
- ☐ No
- ☐ I don’t know
- If Yes, where does the tank drain to?
  - ☐ Open drain
  - ☐ Sewer
  - ☐ Soak pit
  - ☐ I don’t know
  - ☐ Other (specify): _________________________________

9. Provide any additional information about the containment:

____

Description of toilet technology

10. What type of toilet(s) feed into this containment system? (select all that apply)

- ☐ Cistern flush toilet
- ☐ Pour-flush toilet
- ☐ Dry toilet
- ☐ Urine-diverting toilet

- ☐ Wastewater and solid waste

11. What type of anal cleansing material is used? (select all that apply)

- ☐ Toilet paper
- ☐ Water
- ☐ Other (specify): ___________

12. Is anything added to the containment to help with smell or improved degradation?

- ☐ Yes
- ☐ No
- ☐ I don’t know
- If Yes, what is added?
  - ☐ Bio additives/enzymes
  - ☐ Lime
  - ☐ Ash
  - ☐ Other (specify):_________________
- Specify brand name if possible: _________________________
- How often is it added, and how much is added every time?

  ____

13. Are cleaning chemicals disposed of into the toilet?

- ☐ Yes
- ☐ No
- ☐ I don’t know
- If Yes, what chemicals? Specify brand names if possible:

  ____

14. Does solid waste enter the faecal sludge containment system?

- ☐ Yes
- ☐ No
- ☐ I don’t know
- If Yes, what type(s) of solid waste? ________________________________

15. What types of wastewater enter the containment system? (select all that apply)

- ☐ Toilet
- ☐ Bathing
- ☐ Laundry
- ☐ Kitchen
- ☐ I don’t know

Water accessibility

16. Is there a water connection on the premises?

- ☐ Yes
- ☐ No
- ☐ I don’t know

17. Where do you get your water?

- ☐ Tap inside building
- ☐ Standpipe outside building
- ☐ Other (specify): ___________________________
- ☐ I don’t know

Quality of construction

19. How old is the containment?

- ☐ Less than 1 year old
- ☐ 1-5 years old
- ☐ 6-10 years old
- ☐ More than 10 years old
- ☐ I don’t know

20. Who constructed your containment system?

- ☐ Professional engineer
- ☐ Technician/mason
- ☐ Myself, or family/friends
- ☐ I don’t know

Accumulation rate

22. When was the system last emptied? __________________________

23. Was it fully emptied at that time?

- ☐ Yes
- ☐ No
- ☐ I don’t know
- ☐ System has never been emptied

24. What is the typical emptying interval of your containment system?

- ☐ Every ______ years (specify)
- ☐ I don’t know

References

(1) Andriessen, N.; Strande, L. *Volaser: A Faecal Sludge Measuring Device - Assembly and Users Guide*; Swiss Federal Institute for Aquatic Science and Technology (Eawag): Dübendorf, Switzerland, 2023. https://www.eawag.ch/fileadmin/Domain1/Abteilungen/sandec/publikationen/EWM/Volaser/volaser_manual.pdf.

(2) *Standard Methods for the Examination of Water and Wastewater*, 22nd ed.; Rice, E. W., Baird, R. B., Eaton, A. D., Clesceri, L. S., Eds.; Bridgewater, L., Series Ed.; APHA; AWWA; WEF: Washington, D.C., 2017.

(3) *Methods for Faecal Sludge Analysis*, 1st ed.; Velkushanova, K., Strande, L., Ronteltap, M., Koottatep, T., Brdjanovic, D., Buckley, C., Eds.; IWA Publishing: London, UK., 2021. https://doi.org/10.2166/9781780409122.

(4) Semiyaga, S.; Okure, M. A. E.; Niwagaba, C. B.; Nyenje, P. M.; Kansiime, F. Dewaterability of Faecal Sludge and Its Implications on Faecal Sludge Management in Urban Slums: Faecal Sludge Pre-Treatment by Dewatering. *Int. J. Environ. Sci. Technol.* **2017**, *14* (1), 151–164. https://doi.org/10.1007/s13762-016-1134-9.

(5) Leverenz, H. L.; Tchobanoglous, G.; Darby, J. L. *Evaluation of Greenhouse Gas Emissions from Septic Systems*; DEC1R09; Water Environment Research Foundation & IWA Publishing: Virginia, US, 2011. https://doi.org/10.2166/9781780403359.

(6) Huynh, L. T.; Harada, H.; Fujii, S.; Nguyen, L. P. H.; Hoang, T.-H. T.; Huynh, H. T. Greenhouse Gas Emissions from Blackwater Septic Systems. *Environ. Sci. Technol.* **2021**, *55* (2), 1209–1217. https://doi.org/10.1021/acs.est.0c03418.

(7) Poudel, P.; Ghimire, A.; Howard, G.; Evans, B.; Camargo-Valero, M. A.; Mills, F.; Reddy, O.; Sharma, S.; Tuladhar, S.; Geremew, A.; Okurut, K.; Ngom, B.; Baidya, M.; Dangol, S. Field-Based Methods for Measuring Greenhouse Gases Emissions from on-Site Sanitation Systems: A Systematic Review of Published Literature. *Heliyon* **2023**, *9* (9), e19947. https://doi.org/10.1016/j.heliyon.2023.e19947.

(8) Xie, H.; Zuo, X.; Chen, Y.; Yan, H.; Ni, J. Numerical Model for Static Chamber Measurement of Multi-Component Landfill Gas Emissions and Its Application. *Environ Sci Pollut Res Int* **2022**, *29* (49), 74225–74241. https://doi.org/10.1007/s11356-022-20951-2.

(9) Diaz-Valbuena, L. R.; Leverenz, H. L.; Cappa, C. D.; Tchobanoglous, G.; Horwath, W. R.; Darby, J. L. Methane, Carbon Dioxide, and Nitrous Oxide Emissions from Septic Tank Systems. *Environ. Sci. Technol.* **2011**, *45* (7), 2741–2747. https://doi.org/10.1021/es1036095.

(10) Andriessen, N.; Appiah-Effah, E.; Browne, S. J. I.; al Jahjah, R.; Kabika, J.; Kinobe, J. R.; Korir, N.; Nishimwe, P.; Niwagaba, C. B.; Pradeep, R.; Prasad, P.; Tembo, J. M.; William, A.; Ambuehl, B.; Strande, L. Quantities and Qualities of Fecal Sludge: Experiences from Field Implementation with a Volaser in 7 Countries during a Pandemic. *Front. Water* **2023**, *5*, 1130081. https://doi.org/10.3389/frwa.2023.1130081.

(11) Khatun, S.; Berg, J. S.; Jézéquel, D.; Moiron, M.; Escoffier, N.; Schubert, C. J.; Bouffard, D.; Perga, M.-E. Long-Range Transport of Littoral Methane Explains the Metalimnetic Methane Peak in a Large Lake. *Limnology and Oceanography* **2024**, *69* (9), 2095–2108. https://doi.org/10.1002/lno.12652.

(12) Strande, L.; Schoebitz, L.; Bischoff, F.; Ddiba, D.; Okello, F.; Englund, M.; Ward, B. J.; Niwagaba, C. B. Methods to Reliably Estimate Faecal Sludge Quantities and Qualities for the Design of Treatment Technologies and Management Solutions. *Journal of Environmental Management* **2018**, *223*, 898–907. https://doi.org/10.1016/j.jenvman.2018.06.100.
